# Questioning the preclinical paradigm: natural, extreme biology as an alternative discovery platform: A New Modest Proposal for Preventing the Children of the World from Being Burdened, Like Their Parents, by Disease and Making Beneficial Therapies (shamelessly co-opted from Dr. Swift)

**DOI:** 10.18632/aging.100704

**Published:** 2014-12-04

**Authors:** Rochelle Buffenstein, O. Lynne Nelson, Kevin C. Corbit

**Affiliations:** ^1^ Barshop Institute for Aging and Longevity Studies, University of Texas Health Science Center at San Antonio, TX University of Texas Health Science Center at San Antonio, TX USA; ^2^ Department of Veterinary Clinical Sciences, Washington State University, Pullman, WA 9916, USA

**Keywords:** preclinical models, extremophiles, grizzly bear, insulin sensitivity, obesity, cardiac function, naked mole-rat, aging, cancer resistance

## Abstract

The pace at which science continues to advance is astonishing. From cosmology, microprocessors, structural engineering, and DNA sequencing our lives are continually affected by science-based technology. However, progress in treating human ailments, especially age-related conditions such as cancer and Alzheimer's disease, moves at a relative snail's pace. Given that the amount of investment is not disproportionately low, one has to question why our hopes for the development of efficacious drugs for such grievous illnesses have been frustratingly unrealized. Here we discuss one aspect of drug development –rodent models – and propose an alternative approach to discovery research rooted in evolutionary experimentation. Our goal is to accelerate the conversation around how we can move towards more translative preclinical work.

“My iPhone just told me I turn 86 today. Do you think you can hurry up with that cure?” – Jean Corbit, grandmother of the corresponding author

## The Current Preclinical Paradigm

For more than a century, most biomedical research has relied primarily on mice and rats to study the basic biology, progression, and prevention of disease, with the overarching premise that “below the skin” all organisms are molecularly and biochemically alike. Indeed, several seminal discoveries and human therapies have been made using the premise of rodent models. However, advances in certain areas, especially age-related diseases, have been slow. In fact, one can argue that the numerous reported ‘cures’ for rodent obesity, cancer, and Alzheimer's disease have ultimately burdened the collective resources of the community to the point that a re-evaluation of the preclinical paradigm must be undertaken.

With the advent of transgenic rodent technologies, a revolution of new discoveries and ideas was ushered in. Particularly in the field of Developmental Biology, both loss- and gain-of-function genetic experiments elucidated many of the major pathways governing gastrulation, patterning, and embryonic development in general. Indeed, so stereotypical is vertebrate development that to the untrained eye it can be difficult to discern early mouse and human embryos.

However, animals diverge developmentally, behaviorally, and metabolically postpartum, most notably in adulthood. Because of this the applicability of rodent disease models has been called into question (e.g. see the European Commission workshop report at http://ec.europa.eu/research/health/pdf/summary-report-25082010_en.pdf and [1]). This is particularly evident in diseases such as obesity, where aberrations in body weight are appreciated in ~1/3 of mouse knockouts [2]. Indeed, almost every developmental pathway – BMP [3], TGFβ[4] FGF [5], VEGF [6], Hedgehog [7], Wnt [8], and Notch [9] – have been reported to be anti- or pro-obesogenic. It is difficult to rationalize why mammals would evolve such that perturbation of any signaling pathway would affect body mass, especially weight loss.

Specifically, the issue is not ‘reverse translation’ – drugs that are clinically successful in humans having effects in rodents -in fact many do [1], but instead the many ‘cures’ reported in mice fail once reaching human clinical trials. It is difficult to fully appreciate the magnitude of the problem until being immersed in the world of drug development, possibly due to a lack of appropriate publication outlets for such ‘negative data’. With such disparity between mouse and human efficacy, major biotechnology and pharmaceutical companies have invested heavily in human genetics (e.g. see http://wwwext.amgen.com/media/media_pr_detail.jsp?releaseID=1765710 and http://www.nytimes.com/2014/01/13/business/aiming-to-push-genomics-forward-in-new-study.html?_r=0). With success stories such as PCSK9 [10, 11] the hope is to shine a spotlight on high-value targets in a sea of positive data derived from rodents.

### The Workhorse of Discovery Biology

One mouse strain in particular, C57Bl/6, predominates studies to investigate molecular mechanisms of disease and evaluate putative therapies (for a robust commentary read Daniel Engber's piece in Slate: http://www.slate.com/articles/health_and_science/the_mouse_trap/2011/11/black_6_lab_mice_and_the_history_of_biomedical_research.html). There are many advantages to using this well characterized and highly inbred mouse strain, most notably these rodents are amenable to genetic manipulation and acquired data using this reductionist approach are highly reproducible. Moreover mice rapidly attain sexual maturity, have good breeding success in captivity and develop many of the diseases that commonly afflict humans. As such, it is inferred that experimental findings in C57Bl/6 mice apply to all mammals including humans. Disappointingly, many of the promising preclinical findings based upon lab rodents have not translated into effective and safe biomedical discoveries for human therapies [12]. This may reflect, in part, that these genetically engineered mice have been selected for rapid growth, cancer susceptibility and short-lives and naturally have poor defenses against environmental toxins and disease. Supporting this premise are findings that C57Bl/6 mice live only half as long as predicted on the basis of body size [13] and have shorter lifespans than their more genetically diverse wild-caught counterparts [14].

Another possible explanation for their short-lifespans and early onset of age-associated diseases may be linked to the animal husbandry conditions. In most research facilities mice are maintained in a manner geared towards the comfort and convenience of the human animal-attendants such that rodents are housed in temperature- and light-controlled, specific pathogen free facilities. Furthermore, they are kept in small cages, which restrict exercise and access to copious enrichment. These germ-free animals exhibit artificial neuroendocrine rhythms, do not have an inbuilt library of antibodies to deal with pathogens, and are very vulnerable to environmental stressors. Also, animals with little mental or physical stimulation often become obese, express different proteome profiles from exercised animals, and may develop a suite of pathological conditions that may constrain or confound the pertinence of research based findings [15]. Finally, mice living below thermoneutrality (21-24°C) expend half of their daily energy expenditure on facultative thermogenesis alone and, as a result, chronically cold-stressed mice eat twice as much as mice housed at 28-30°C [16]. Normalizing metabolic homeostasis in various models using such hypermetabolic animals may be setting the bar a bit low.

Data obtained from animals housed individually or in groups in which they can huddle together and behaviorally thermoregulate, especially with regard to drug treatments, markedly differ. For example, we have found, upon examining cardiac responses to doxorubicin, a widely used chemotherapy with concomitant cardiac toxicity, animals housed on their own fare considerably worse than those housed in groups (Grimes and Buffenstein, unpublished data). Moreover, mice housed under standard sub-thermoneutral temperatures are more susceptible to tumor growth [17].

It has been reported that *ad libitum* fed, sedentary mice exhibit reduced cognitive function and commonly present with similar pathophysiology to those of morbidly obese humans [18, 19]. Astonishingly, these individuals would be commonly considered “unhealthy” by human criteria yet are routinely used as experimental “controls” with which to evaluate the efficacy of a treatment or experimental intervention. Not surprisingly, interventions that reduce food intake (i.e. dietary restriction) or enable the animals to increase energy expenditure through exercise or participate in mentally challenging activities commonly show beneficial and life-extending effects [20-23]. Conversely, if these sedentary rodents are subjected to high fat diets or other sub-optimal husbandry conditions they commonly become even more susceptible to oxidative stress and glucose dysregulation, and as such may be more responsive to experimental manipulations to reduce oxidative stress, or drugs that treat metabolic syndrome [24, 25].

## Evolutionary Experimentation as a New Paradigm

There is a growing call for additional discovery tools in biomedical research that provide more translative predictability for diseases that generally afflict humans in later life. Animal models that are considered long-lived on the basis of their body size are essential to fill the gap assessing the immutable role of time in aging and the manifestation of age-related diseases. Use of extremely long-lived models such as the naked mole-rat, [26] or species that have adapted to extreme environments [27-29] also enables one to evaluate whether nature has already evolved the appropriate mechanisms to overcome the environmental threats that contribute to sporadic and late onset diseases.

An alternative approach towards target discovery employs natural, extreme biology where evolutionary experimentation has overcome many biological challenges [30-33]. For instance, obesity is a natural and necessary state to survive months of fasting in hibernating animals. To this end we studied grizzly bears (*Ursus arctos horribilis*) before, during, and after hibernation to determine the effects of natural obesity on insulin sensitivity and cardiac function.

## The Metabolic Adaptations Governing Hibernation

Mammalian hibernation is a distinct adaptive strategy that intrigues scientists due to an impressive display of extreme and flexible metabolism. Hibernation is most commonly defined as a series of physiological alterations in various tissues that give an animal the ability to survive long periods of nutritional deprivation by dramatically reducing energy metabolism [34]. The sum of these adaptations results in a radical departure from the normal physiological homeostasis that is seen in most mammals. Numerous species, including primates, manifest a torpid phenotype linked to nutrient availability or thermal challenges, although the characteristics of the hypometabolic state can be quite different among estivators and hibernators, and even within both small- and large-sized hibernators marked interspecific differences are evident.

Ground squirrels *(Spermophilus spp.)* are often considered the prototypical hibernator as individuals in this genus transition from an active, euthermic state (37^o^C) to a hibernating state where torpid body temperature commonly falls to 3-5^o^C. During the hibernation cycle, these animals may be completely inactive for weeks at a time at near freezing body temperatures, but exhibit repeated arousal bouts to euthermy lasting approximately 24 hours [35-38]. Ground squirrels are nonresponsive during bouts of torpor, but awaken during periodic interbout arousals. In contrast, the larger species of the genus *Ursus* maintain a considerably more homogenous hibernation state than ground squirrels, as bears maintain near normal body temperatures and cyclic interbout arousal periods are absent [39, 40]. In general, bears are not unconscious and may be easily disturbed during hibernation, often appearing quite alert [41-43]. Bears hibernate for 4-6 months without nutritional intake, whereas many small hibernators arouse every 4-10 days to feed [44, 45]. As such, bear hibernation may represent the most refined response to dormancy of any mammal.

### Three states of insulin sensitivity in bears

To cope with the food scarcity of winter many mammals enter a hyperphagic period in late summer and early autumn to store sufficient fat necessary to fuel months of fasting. During this preparatory phase bears double total body fat and increase body weight by 30-50%. To understand the metabolic consequences of such acute fat and weight accumulation, we assayed insulin responsiveness in bears during three seasons: autumn (pre-hibernation in October when the bears are most obese), winter (hibernation in January), and spring (April/May following arousal from hibernation but before mating or hyperphagia). We uncovered three distinct states of insulin sensitivity – a typical response in spring, augmented sensitivity in autumn, and resistance during hibernation [46].

Interestingly, the increased insulin sensitivity observed in obese bears preparing for hibernation occurs selectively in adipose tissue. Specifically, bears shut down the lipid phosphatase PTEN to promote AKT activity and heighten insulin responsiveness. This is at least partially governed by a phosphorylation event on serine 380 (S380) of PTEN that is not observed in other tissues such as liver and skeletal muscle, suggesting the intriguing possibility of an adipose tissue-specific PTEN S380 kinase that could be a target to develop insulin-sensitizing drugs. Work demonstrating that *PTEN* haploinsufficient humans are exquisitely insulin responsive and simultaneously obese makes them not only similar to autumn bears, but also supports the existence of an evolutionarily conserved mechanism of adipose tissue-specific insulin sensitization [47]. We propose that bears developed reversible insulin resistance to govern fat metabolism; augmented adipose tissue insulin sensitivity in autumn to promote fat storage and hibernation-specific insulin resistance to mediate lipolysis and obligate use of fat as the predominant fuel source to survive prolonged fasting. Our results using natural, extreme biology add to an emerging paradigm where diabetes and obesity - in contrast to the prevailing notion that the two always go hand-in-hand - may exist naturally on opposite ends of the metabolic spectrum. Finally, given that the amount of circulating insulin does not change during the sensitive-resistant cycle and bears exist with relatively low levels of insulin, rigorously testing the idea of *lowering* insulin levels to treat diabetes and obesity may have evolutionary rationale [48-51].

### Cardiac adaptions during hibernation

Both ground squirrels and bears dramatically reduce heart rate and cardiac output during hibernation relative to the active state. However, divergent cardiac phenotypes and adaptive strategies are manifest by the two species. For example, grizzly bears maintain a normal stroke volume while reducing cardiac muscle mass and muscle compliance during hibernation, opposite to what is observed in ground squirrels [52-54]. The maintenance of stroke work with smaller cardiac muscle mass by the bear is unique in that chronic, prolonged diastolic filling is typically associated with eccentric remodeling (dilation) resulting in larger muscle mass. However, a chamber that increases in stiffness is less likely to distend or dilate. Indeed, a part of the bears' adaptation to hibernating at very low heart rates is to alter the characteristics of the cardiac muscle. Upregulation of the stiff sarcomeric protein, titin (N2B isoform), compensates for the reduced cardiac muscle mass by increasing muscle stiffness and normalizing ventricular wall stress with associated with prolonged diastolic filling at low heart rates. [55] Correlations between titin expression and ventricular diastolic function have been observed in humans [56, 57]. In dilated cardiomyopathy, increased titin N2BA isoform expression results in a decrease in ventricular stiffness, which may be important in reducing ventricular filling pressures for a given volume (reducing symptoms of congestion) [58]. However, increased N2BA expression has been correlated with a negative effect on systolic function and worsening heart failure [56, 59]. Understanding the mechanisms that allow for preserved function yet maintenance of a lower muscle mass in an energetically costly organ such as heart may constitute important treatment strategies to reduce overall cardiac workload in disease.

Throughout their evolutionary history, bears have naturally solved ways to avoid the negative consequences of low cardiac output and heart rate, and yet remain homeothermic and responsive. Given that the protein and gene regulations presently identified in hibernation are not novel and are shared by all other mammals, what are the potential applications of this physiology to non-hibernating mammals? Comparative physiologic studies involving some form of biomimcry could result in improved treatment strategies for cardiac disease in humans.

## Extremophiles, Aging, and Peto's Paradox

The fascinating biology of the grizzly bear's evolved armory against the vagaries of hibernation serves to remind us that many seminal discoveries in biomedical sciences have been serendipitously found employing unusual animal models. Comparative physiological and evolutionary studies involving animals inhabiting extreme habitats has repeatedly shed light on elusive biomedical questions and basic biology. Not surprisingly, some of these “extremophiles” (a term used more loosely here than to simply refer to members of the domains Archea and Bacteria, microbes known to live in particularly inhospitable habitats) have been co-opted into the “traditional” biomedical animal model bestiary.

### C. elegans as an idiosyncratic model organism for biomedical research

One such species is *Caenorhabditis elegans*, a soil-dwelling microscopic, hermaphroditic roundworm. *C.elegans* shares multicellular complexity and signaling pathways with higher organisms. This together with its short-lifespan, ease of standardized husbandry and genetic manipulation have rendered it a particularly useful model for high throughput functional genomic screens of novel drug targets [60]. *C.elegans* has led to significant findings regarding the highly conserved insulin signaling pathway among metazoans [61] and in particular the role thereof in longevity [62, 63]. Over the last fifty years, every biological aspect of this unsegmented pseudocoelomate has been extensively studied, providing fundamental insights into developmental biology, genomics, neurobiology, and aging (see http://www.wormbook.org/ *NCBI)*. Indeed, research based upon this unusual worm has resulted in three Nobel prizes for Medicine.

### Methuselah models for aging research

In aging studies among mammals two species stand out as being exceptionally long-lived, namely, naked mole-rats and humans, both living ~5 times longer than predicted allometrically. This mouse-sized, 30 year-living rodent exhibits very few age-associated declines, maintaining body composition, heart health, bone quality, and reproductive capacity for at least 24 years [26, 64-66]. Strikingly, the naked mole-rat, although it does not employ torpor, shares many similar physiological features with those of hibernating mammals; both basal heart rate and metabolic rate are lower than predicted on the basis of body size [65]. Echocardiography revealed that all left ventricular functional parameters were substantially lower than those of mice indicating that their hearts are “idling on low” and border on levels considered to represent pathological left venticular systolic dysfunction in humans [67]. Moreover, naked mole-rats show extreme tolerance to pronounced thermolability as well as oxygen and nutrient availability [68-71], features considered highly adaptive to life under the extreme conditions encountered in xeric subterranean habits. Another adaptive feature to life in arid habitats are low fasting blood glucose levels and a reliance on volatile fatty acids, derived from the fermentation of cell walls by gut microbiota, as a predominant fuel source [72]. Finally, following both grizzly bears and the aging paradigm in general, naked mole-rats are exquisitely sensitively to and have very low circulating levels of insulin [73, 74].

### Cancer resistant animal models

Another unusual characteristic of naked mole-rats, shared with much larger bears and blue whales [75], is that, unlike most mammals, cancer is extremely rare. Naked mole-rat differences in cancer susceptibility are attributed to greatly enhanced stringency for proliferative control (e.g., p53, pRb, and the Ras/MAPK pathway) and over-expression of genes both encoding products that protect DNA during transcription and promote xenobiotic detoxification [76-79].

Indeed, in most multicellular animals, incidence of cancer ranges between 20-50% [80, 81], regardless of phylogeny, body size and the evolution of multiple defense mechanisms to protect genomic stability. Mutational frequency is known to be similar in cells of all species and, as such, the accrual of mutations ought to depend upon the number of cell divisions, rendering larger species (e.g. blue whales) with a greater number of cells more prone to malignancy the smaller animals (e.g. mice). Moreover, although long-lived species intuitively should exhibit more DNA mutations by the end of life than observed in short-lived species, cancer incidence generally is the same or greater in short-lived species [82, 83], a phenomenon commonly referred to as Peto's paradox [84]. As such, although most elderly individuals have numerous cells with the requisite mutations necessary for cancer initiation, the progression into invasive cancer is relatively rare. This is not attributable to efficient DNA repair pathways; rather it has been proposed that extraneous factors within the cellular milieu disturb normal cell signalling pathways, promoting cancer progression and metastasis and may be directly linked to metabolic profiles [81]. Given the critical role of the metabolic milieu in cancer progression, data acquired from cancer cells maintained under optimal culture conditions may have limited translational potential, findings that have been confirmed in assessments of effective drug treatments [85]. Similarly, experimentally induced cancer initiation in highly inbred mouse models may not be ideal models for the complexity of human cancer progression and metastasis [86].

A common feature shared by species that exhibit a relatively low rate of cancer incidence is comparatively low mass-specific metabolic rates. Altered cellular metabolism, particularly in response to glucose dysregulation and obesity, has been strongly implicated in multiple human cancers [87]. Indeed, an altered cancer risk in immigrants taking on a Westernized lifestyle has been appreciated [88]. Lack of physical activity coupled with obesity and associated changes in circulating levels of insulin, IGF, mTOR and associated kinases have been strongly implicated in this regard. Indeed it appears that a positive energy balance and the anabolic metabolic pathways involved in this regard play a dominant role in the severity of cancer progression. While this evolutionarily conserved pathway is essential for synchronizing growth and repair to match energy availability and thereby ensure survival of the organism, many of the intermediates in this nutrient sensing pathway are tumor suppressor proteins (e.g., PTEN and LKB1). Chronic activation of PI3 kinase signaling, by elevated blood glucose in diabetes or metabolic syndrome, may thus increase the risk of the development of cancer, leading to a metabolic switch favoring aerobic glycolysis and cell proliferation [89, 90]. Preternaturally cancer-resistant naked mole-rats have naturally have low insulin and mTOR signaling [91] that may protect them against tumorigenesis. Similarly, very few incidences of cancer have been reported in grizzly bears, or even bears in general [92-94], that, like the naked mole-rat, may downregulate mTOR signaling, albeit seasonally [46]. Understanding how environmentally induced changes in neuroendocrine signaling influence proliferative phenoplasticity and prevent age-associated diseases commonly linked to metabolic dysfunctions may lead to paradigm shifts in our approaches to targeted drug discovery. The paramount question is, however, if what is ultimately discovered from evolved natural cancer resistance can be applied to the reversal of established cancers in humans.

## Closing remarks

In 1950, C. Ladd Prosser wrote “Comparative Physiology is not so much a defined discipline as a viewpoint, a philosophy”. We believe it is worth examining if this philosophy extends to the discovery of more predictive targets for drug development. When biological findings are rooted in the natural adaptations of evolutionary pressures the governing mechanisms are likely to be conserved, as opposed to when artificial genes, diets, and environments are imposed upon rodents. For instance, when mice lacking an immune system are injected with human tumor cells, or when inbred mice have their left coronary arteries tied off, or obesity-prone mice are fed a human ‘cafeteria’ diet, the reaction from animals never exposed to such insults can be anything but natural. Decades of brilliant science, exciting findings, and substantial investment have far too often not translated when put to the challenge of human clinical trials. An alternative approach utilizes carefully chosen extremophiles that have evolved genes, proteins, and mechanisms to naturally counter disease-inducing insults and ultimately follows Orgel's second rule: “*Evolution is cleverer than you are*”. As a community we need to reconsider the future of preclinical research if we are to obtain broader measures of success in treating the grievous illnesses that face us today.
